# Tandem peptide receptor radionuclide therapy using ^90^Y/^177^Lu-DOTATATE for neuroendocrine tumors efficacy and side-effects - polish multicenter experience

**DOI:** 10.1007/s00259-020-04690-5

**Published:** 2020-01-24

**Authors:** Jolanta Kunikowska, Anna Zemczak, Maciej Kołodziej, Paweł Gut, Izabela Łoń, Dariusz Pawlak, Renata Mikołajczak, Grzegorz Kamiński, Marek Ruchała, Beata Kos-Kudła, Leszek Królicki

**Affiliations:** 1 Nuclear Medicine Department, Medical University of Warsaw, ul. Banacha 1 a, 02-097 Warsaw, Poland; 2 Department of Endocrinology and Neuroendocrine Tumors, Department of Pathophysiology and Endocrinology, Medical University of Silesia, Katowice, Poland; 3 Division of Endocrinology, Department of Pathophysiology and Endocrinology, Medical University of Silesia, Katowice, Poland; 4 Department of Endocrinology and Radioisotope Therapy, Military Institute of Medicine, Warsaw, Poland; 5 Department of Endocrinology, Metabolism and Internal Medicine, Poznan University of Medical Sciences, Poznan, Poland; 6 Department of Internal Medicine, Hypertension and Vascular Diseases, Medical University of Warsaw, Warsaw, Poland; 7 Radioisotope Centre POLATOM, National Centre for Nuclear Research, -Świerk, Otwock, Poland

**Keywords:** PRRT, ^90^Y/^177^Lu-DOTATATE, Tandem therapy, Somatostatin receptor, Neuroendocrine tumors

## Abstract

**Introduction:**

One of the concepts of theranostics in nuclear medicine is peptide receptor radionuclide therapy (PRRT), whereby labeled somatostatin analogs are used for imaging and treating inoperable or disseminated neuroendocrine tumors (NET).

**Aim:**

The aim of the study was to determine the therapeutic efficacy and toxicity of tandem ^90^Y /^177^Lu-DOTATATE in patients with disseminated NET in a multicenter trial.

**Materials and methods:**

103 patients with NET G1/G2 treated with ^90^Y/^177^Lu-DOTATATE (1:1) with amino-acid infusion for nephroprotection were included in the study.

**Results:**

Overall survival from the disease diagnosis (OS-D) was 127.4 months and from the time of PRRT (OS-T) was 89.5 months. Progression-free survival (PFS) was 29.9 months. An analysis based on the proliferation index revealed a statistically significant impact on PFS and OS-T (PFS G1 vs G2, 59.3 vs 24.3 months; OS-T G1 vs G2, not reached vs 79.9 months). The effect of the primary disease site was also analyzed. For pancreatic vs small bowel vs large bowel, the PFS was 30.8 vs 30.3 vs 40.6 months, the OS-T was 94 vs 61.9 vs 131.2 months and OS-D was 130.4 vs 89.2 vs not reached months, respectively. The 2-year risk of progression was 42%. The probability of 2-year and 5-year overall survival was 89% and 62%, respectively. PRRT was well tolerated by all patients. One patient (1%) developed myelodysplastic syndrome. No other grade 3 and 4 hematological or renal toxicity was observed.

**Conclusions:**

This multicenter trial showed that tandem ^90^Y/^177^Lu-DOTATATE is highly effective and safe therapy for patients with disseminated NET.

## Introduction

The term theranostics in nuclear medicine refers to combined diagnostic imaging and therapy using the same molecule. One of the most successful examples of the theranostics concept in nuclear medicine is peptide receptor radionuclide therapy (PRRT) for imaging and treatment of well differentiated neuroendocrine tumors (NET).

Neuroendocrine tumors are a heterogeneous group of neoplasms, arising from cells of the endocrine system, with various clinical behaviors. Although they may show hormonal activity (known as hormonally active tumors), a significant proportion do not produce enough hormones and/or biogenic amines to cause clinical symptoms (classed as hormonally inactive tumors) [1–4]. Although these neoplasms are considered rare, a significant increase in the incidence and detectability of NET has been noted in many epidemiological studies in recent years [2–5].

Among many options for pharmacological treatment of these tumors, long-acting somatostatin analogs which not only reduce symptoms of the disease but also have anti-proliferative effects [6, 7] remain the mainstay therapy. For patients with inoperable disease or whose disease progresses despite long-acting somatostatin analog therapy, PRRT is a reasonable second-line approach. Confirmation of somatostatin receptor overexpression by nuclear medicine imaging [1, 8] is a prerequisite for patient selection.

Initially, trials with [^111^In-diethylenetriaminepentaacetic acid (DTPA)^0^]-octreotide showed limited patient responses which were, in part, related to the physical characteristics of ^111^In [9, 10].

Higher response rates were obtained using the β-emitters, ^90^Y-DOTA0-Tyr3–octreotide (^90^Y-DOTATOC) and ^177^Lu-DOTA0-Tyr3–octreotate (^177^Lu-DOTATATE), which had a greater impact on tumor volume due to superior tissue penetration. Studies assessing the efficacy of ^90^Y-DOTATOC showed a favorable response to treatment in 10–34% of patients [11–13].

Due to renal excretion, the high maximum energy ([E_max_] 2.27 MeV) and a long maximum particle range (10 mm) beta-particle emission by ^90^Y with has been shown to cause significant nephro toxicity. In contrast, ^177^Lu emits lower energy ([E_max_] 0.497 MeV) and shorter particle range (maximum 2–4 mm) beta particles, and is an alternative to ^90^Y. The clinical results of [^177^Lu-DOTA^0^,Tyr^3^,Thr^8^]octreotate (DOTATATE) therapy showed a very promising therapy response rate, with mild bone marrow suppression and low nephrotoxicity [14, 15]. The impact of this type of treatment on overall survival (OS) of patients with NET has been shown in a prospective, randomized, phase III trial evaluating the effect of ^177^Lu-DOTATATE in combination with octreotide LAR 30 mg vs octreotide LAR 60 mg in midgut tumors [16].

At the same time, attempts were made to combine the complementary characteristic of the beta emitters with a long and short range of action to improve treatment of tumors of various sizes. In a rat model studied, by De Jong et al., a threefold increase in survival was demonstrated when using simultaneous a mix of 50% ^177^Lu-DOTATATE and 50% ^90^Y-DOTATOC [17, 18].

Based on these results, we decided to check the effects of simultaneous ^90^Y/^177^Lu-DOTATATE (tandem therapy) in humans. The comparison of simultaneous ^90^Y/^177^Lu-DOTATATE and ^90^Y-DOTATATE alone was investigated, showing prolongation of OS in the group of patients treated with tandem therapy, with a comparable safety level of both methods [19]. The data of single center trial using simultaneous ^90^Y/^177^Lu-DOTATATE was published in 2017 [20]. These results enabled inclusion of PRRT using ^90^Y/^177^Lu-DOTATATE tandem therapy to the treatment protocols used in Poland and the recommendations of the Polish Network of Neuroendocrine Tumors [1, 2, 21].

In the literature, it is limited data on use simultaneous ^90^Y/^177^Lu-DOTATATE. To confirm our previous results, we examine efficacy of simultaneous ^90^Y/^177^Lu-DOTATATE in lager group of patients in multicenter trial.

**The aim** of the present work was in a multicenter trial to determine the therapeutic efficacy and toxicity of the simultaneous combination treatment, tandem ^90^Y /^177^Lu-DOTATATE, in patients with disseminated NET. The main endpoints were progression-free survival (PFS), OS, and treatment safety. Additionally, we assessed survival parameters relative to disease grading and primary tumor location.

## Material and methods

This was a multi-institution study approved by the ethical committees of Medical University of Warsaw, Military Institute of Medicine, Warsaw, and University of Medical Sciences, Poznan. We retrospectively analyzed a consecutive cohort of patients who had well-differentiated NET with Ki-67 index ≤20% and underwent tandem ^90^Y /^177^Lu-DOTATATE therapy. All patients gave written informed consent.

### Patients

103 patients (39 males and 64 females at the mean age (± SD) of 56.7 ± 11.5 years) with diffused, histologically confirmed NET were included in the study.

Tumors were categorized according to the current TNM staging and grading system for NET.

All patients showed the following inclusion criteria:

- Histological confirmation of NET G1 or G2 tumor; metastatic, inoperable disease.
- Preserved hematological, liver and renal parameters: hemoglobin ≥10 g/dL, white blood cell (WBC) count ≥3 × 10^9^/L, platelet count ≥90 × 10^9^/L, bilirubin ≤1.5 × upper limit of normal (ULN), ALT <2.5 × ULN, and estimated creatinine clearance (CrCl) > 40 mL/min.
- Positive somatostatin receptor imaging (SRI): PET/CT using ^68^Ga-DOTATATE or scintigraphy using ^99m^Tc-HYNICTOC (Tektrotyd, POLATOM, Poland), with Krenning score ≥ 2/3.
- Karnofsky index ≥70, ECOG performance status ≤2.
- Age > 18 years.
- Life expectancy >3 months.
- No pregnancy or lactation.

The study was conducted in accordance with the principles of the Declaration of Helsinki and Good Clinical Practice guidelines.

### Study treatment and radiopeptide administration

All patients received simultaneous intravenous infusions of a mix of ^90^Y/^177^Lu-DOTATATE, comprising 50% radioactivity of ^90^Y-DOTATATE and 50% radioactivity of ^177^Lu-DOTATATE (administered activity per one cycle: 3.7 GBq: 1.85 GBq ^90^Y-DOTATATE +1.85 GBq ^177^Lu-DOTATATE or 2.96 GBq: 1.48GBq ^90^Y-DOTATATE +1.48-GBq ^177^Lu-DOTATATE).

As 2.5% arginine and 2.5% lysine solution was not available, a 1000 mL of mixed amino acid infusion (1000 mL, Vamin 18 Fresenius Kabi, Aminomel 12,5 Baxter, Nephrotec 10%, Fresenius Kabi) and Ringer’s solutions (500 mL) were used for renal protection, 200 mL over 30–60 min immediately before the therapy, continued during the therapy and for 8 h after the therapy [22].

Before administration of the radiopharmaceutical, ondansetron (8 mg, Zofran, Glaxo Wellcome, Atossa, Anpharm SA.) was given intravenously to prevent nausea and vomiting.

The tandem therapy ^90^Y/^177^Lu-DOTATATE was prepared as previously described using ^90^Y and ^177^Lu (ItraPol, LutaPol, POLATOM, Poland) [19, 20]. In patients receiving long-acting somatostatin analog therapy, PRRT was performed 4–5 weeks after injection of octreotide (Sandostatin LAR; Novartis) and 5–7 weeks after injection of lanreotide (Somatuline Autogel; Ipsen). Somatostatin analogue therapy was continued between PRRT cycles. The interval between completing of chemotherapy and PRRT was longer than 3 months.

### Post-therapy imaging

Post-therapy imaging was performed 24 h after the therapy to assess biodistribution. The acquisition was made with an energy window ±10% centered on ^177^Lu photopeaks (208 keV), as described previously [19, 20].

### Assessment of treatment results and clinical benefits

The primary end points were progression-free survival, which was defined as the time from ^90^Y/^177^Lu-DOTATATE treatment to documented disease progression or death from any cause and safety profile. Secondary end points included the objective response rate and overall survival.

All patients underwent staging by contrast-enhanced CT or MRI and SRI using ^99m^Tc-HYNICTOC or ^68^Ga-DOTATATE PET/CT.

Long term response monitoring following PRRT completion was undertaken at 3–6, 12 months and every 12 months thereafter, using blood markers CT or MRI and SRI following the same protocols as had been used prior to therapy.

Blood tests for complete blood cell count and kidney and liver function parameters were repeated every 7–21 days after each therapy cycle, and 3, 6, 12 and every 12 months after completing the therapy. Toxicity was recorded using the Common Terminology Criteria for Adverse Events v3.0 (CTCAE). Kidney function was estimated using the Modification of Diet in Renal Disease formula.

### Statistical methods

Mean values and standard deviations, medians and quartiles or frequencies depending on the parameter distribution were used to summarize patients characteristics.

The progression free survival (PFS) was defined as the time from the start of radioisotope treatment to the first evidence of progression by imaging criteria (SRI and/or CT/MRI).

Overall survival from diagnosis was defined as the time from the first diagnosis of the tumor to death from any cause (OS-D). OS from the start of treatment was defined as the time from the first cycle of ^90^Y/^177^Lu-DOTATATE treatment to death from any cause (OS-T).

OS, PFS, probability of 2-year and 5-year OS, and 2-year risk of progression were calculated using the Kaplan-Meier estimator and compared using the log-rank test [23]. Calculations were performed using GraphPad PRISM 5 (GraphPad Software Inc).

## Results

### Patient characteristics

103 patients underwent PRRT between February 2006 and July 2017.

Of 103 patients, 32 patients were diagnosed with a pancreatic neuroendocrine primary tumor, 29 with a tumor originating from small bowel, 20 with a tumor originating from large bowel, 4 with a bronchopulmonary tumor, 12 had tumors of unknown primary origin and 6 had tumors arising from other sites (1 epiglottis, 1 m**ultiple endocrine neoplasia [**MEN] type 1, 1 von Hippel-Lindau syndrome [VHL] with pancreatic tumor, 2 multiple paragangliomas, 1 retroperitoneal area). 39 patients had low grade tumor NET G1 and 64 had intermediate grade tumor G2 NET.

Prior therapies before commencing PRRT were as follows: 77 patients underwent surgery with primary tumor resection and additionally one patient underwent liver transplantation due to metastases, 75 patients received long-acting somatostatin analog therapy and 23 patients received chemotherapy. Detailed patient data are shown in Table 1. Three patients were lost to follow up and are excluded from further analysis.

**Table 1:** Patient’s characteristics

lp	Patient	Age	Primary orgin	Grading	Surgery	CHT	SSA
1	BR	43	P	2			+
2	BA	39	P	2	+	+	
3	BM	57	LB	2	+		+
4	BM	62	P	2	+	+	+
5	BT	58	SB	2	+		+
6	BM	69	OTHERS (retroperitoneal)	2	+		
7	BJ	45	SB	1	+		
8	CA	72	CUP	1	+		+
9	CO	62	SB	1			+
10	CH	62	P	1	+		+
11	DZ	67	SB	2	+		+
12	DW	50	SB	2	+	+	
13	FD	40	P	2	+		+
14	GK	38	P	1	+		
15	GD	67	LB	1	+		
16	GM	57	SB	2	+		
17	GM	40	SB	2	+		+
18	GS	42	OTHERS (MEN 1)	1	+		
19	GA	47	P	2	+		+
20	JK	26	SB	2	+		+
21	JA	64	SB	2	+		+
22	JR	59	OTHERS (Paraganglioma)	2	+		
23	KB	63	P	2	+		
24	KJ	63	LB	1	+		+
25	KT	44	P	2	+	+	
26	KA	68	LB	1	+		+
27	KM	69	P	2		+	
28	KE	60	SB	2	1		+
29	KM	52	CUP	2			
30	KT	53	LB	1	1		
31	KS	30	CUP	2		+	+
32	KM	57	P	2	+	+	+
33	LA	63	P	2	+		+
34	MK	52	OTHERS (HL)	1	+		
35	MH	72	LB	2	+		+
36	MJ	58	P	2	+		+
37	NG	56	CUP	1	+		
38	NJ	42	LB	2	+		+
39	NL	59	SB	1	+		+
40	NJ	57	SB	1	+	+	
41	PB	37	P	2	+		+
42	PE	45	P	2	+	+	+
43	PM	69	LB	1	+		
44	PC	56	SB	2	+	+	
45	SA	59	LB	2	+	+	+
46	SM	34	SB	2	+		+
47	SE	62	P	2	+		
48	ST	31	P	1	+		+
49	SI	51	P	2	+	+	+
50	SS	54	P	2	+	+	
51	ST	45	CUP	2			+
52	SB	70	SB	1			+
53	SA	52	P	2	+		+
54	ST	46	SB	2			
55	SE	56	SB	1	+	+	
56	SM	33	LB	2	+	+	+
57	SG	65	OTHERS (Paraganglioma)	2	+	+	
58	ŚM	44	SB	1	+		
59	WF	47	OTHERS (epiglottis)	2			
60	WE	54	P	2	+		
61	WG	44	P	2	+		+
62	ZG	54	LB	2	+		
63	ChJ	65	CUP	1		+	+
64	FT	53	P	2		+	+
65	GM	40	CUP	2			+
66	FR	53	LB	1	+		+
67	GW	72	LB	1	+		+
68	GA	61	P	1			+
69	KM	70	CUP	2			+
70	KA	58	P	1			+
71	MM	40	P	1			+
72	WG	60	LB	2			+
73	WB	57	SB	2		+	+
74	WJ	67	SB	1	+		+
75	ZB	68	LB	2	+		+
76	ChK	70	BP	2	+		+
77	FT	67	CUP	2			+
78	KM	62	P	2	+		+
79	RJ	75	LB	1			+
80	SB	70	BP	1		+	+
81	FK	77	CUP	2			+
82	MS	51	P	2	+		+
83	SK	67	BP	1	+		+
84	DC	66	SB	1	+		+
85	PS	73	CUP	1			+
86	WS	70	SB	1	+		+
87	GJ	56	BP	2	+		+
88	J	72	SB	1	+		+
89	KE	62	P	1			+
90	BU	65	SB	1	+		+
91	HW	75	P	1			+
92	BR	65	SB	2	+		+
93	NE	52	P	2		+	+
94	DM	57	LB	2	+	+	+
95	MG	51	LB	2	+		+
96	BM	63	SB	2	+		+
97	AB	71	SB	2	+		+
98	RZ	65	LB	2	+	+	+
99	GŚ	42	P	1	+		+
100	IU	50	LB	1	+		+
101	KD	55	P	2	+		+
102	GL	72	SB	1	+		+
103	KP	65	SB	2	+		+

Tumor primary location: P-pancreas, SB - small bowel, LB- large bowel, CUP – cancer of unknown primary, BP-bronchopulmonary

SSA- long acting somatostatin analogues, CHT- chemotherapy

### ^90^Y/^177^Lu-DOTATATE **tandem therapy**

Patients were usually treated with 4 cycles of 3.7 GBq of ^90^Y/^177^Lu-DOTATATE per injection at 6–12 weeks intervals, depending on clinical status, laboratory results and radiopharmaceutical availability. In patients with hematological or kidney deterioration during treatment or in patients with low body mass (below 50 kg) the injected activity was reduced to 2.96 GBq ^90^Y/^177^Lu-DOTATATE or they received only 3 cycles.

Three patients received 2 cycles, one because of progression during therapy, two because of patient choice. Eight patients received 3 doses with cumulative activity of 11.1 GBq. The remaining patients (89%) completed their planned four cycles of ^90^Y/^177^Lu-DOTATATE treatment with administered cumulative activity of 12.95–14.8 GBq.

All patients with NET-related syndrome received long-acting somatostatin analogs, before and during PRRT with 4–5 weeks after injection of octreotide and 5–7 weeks after injection of lanreotide.

### Imaging response

Imaging response was based on the radiological evaluation according to the Response Evaluation Criteria in Solid Tumors (RECIST) 1.1 criteria and SRI.

It was performed at the first available checkpoint 3–6 months after therapy with partial response (PR) in 17(17%) patients, stable disease (SD) in 78 (78%) patients and progressive disease (PD) in 5 (5%) patients. At 3–6 months follow up complete response (CR) was not observed, no patients died.

Additionally, the imaging evaluation was performed one year after therapy. At one-year follow up eight patients died, CR was seen in 2 (2%) patients, PR in 20 (22%) patients, SD in 59 (64%) patients and PD in 11 (12%)patients.

At 3–6 months vs 12 months follow-up, the disease control rate was 95% vs 88%. The objective response rate (ORR), defined as in the proportion of patients achieving PR or CR, was 17% vs 24%, receptively.

### Survival parameters

The median follow-up duration was 44.1 months (interquartile range IQ: 30.5 to 74.6 months). 41 patients died during follow-up.

Overall survival was calculated for all patients and a Kaplan-Meier survival curve was generated for all evaluable patients in this study. PFS was also calculated (including deaths).

**Fig. 1:**
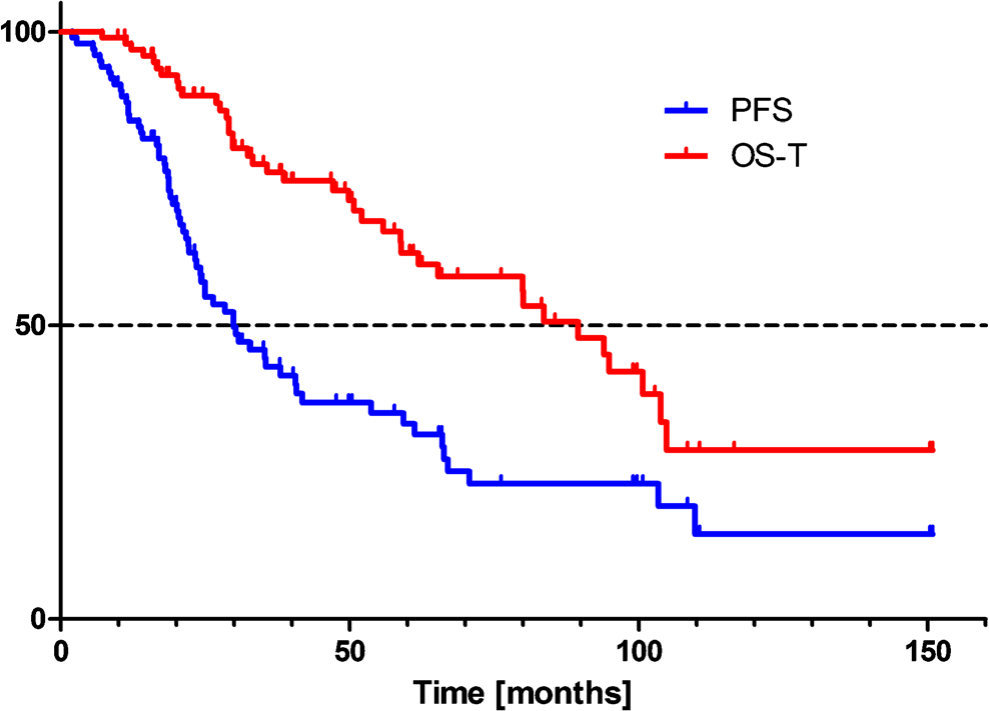
Tandem ^90^Y/^177^Lu-DOTATATE therapy: Kaplan-Meier estimators of overall survival from the time of therapy (OS-T) and progression-free survival

The median overall survival from diagnosis (OS-D) was 127.4 months, and overall survival from the time of PRRT (OS-T) was 89.5 months. PFS was 29.9 months (Fig.1).

The observed 2-year risk of progression was 42%. The probability of 2-year and 5-year overall survival from start of therapy was 89% and 62%, respectively.

### Correlation of grading and survival parameters

When the proliferation index of tumors (G1, *n* = 37; G2, *n* = 63) was taken into consideration, Kaplan–Meier curve analysis showed a significant impact on PFS and OS-T.

**Fig. 2:**
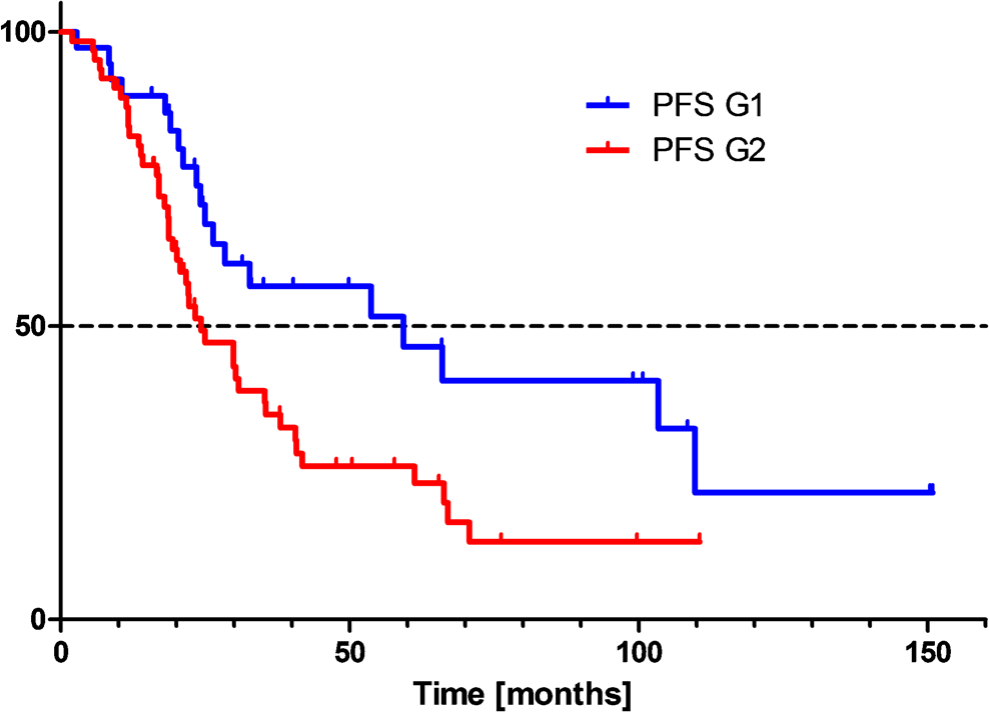
Tandem ^90^Y/^177^Lu-DOTATATE therapy: Kaplan-Meier estimators of progression-free survival in relation to the disease grade

Patients with G1 tumors had a significantly longer PFS, median PFS 59.3 months, compared with patients with G2 tumors, median PFS 24.3 months (*P* = 0.016) (Fig.2).

**Fig. 3:**
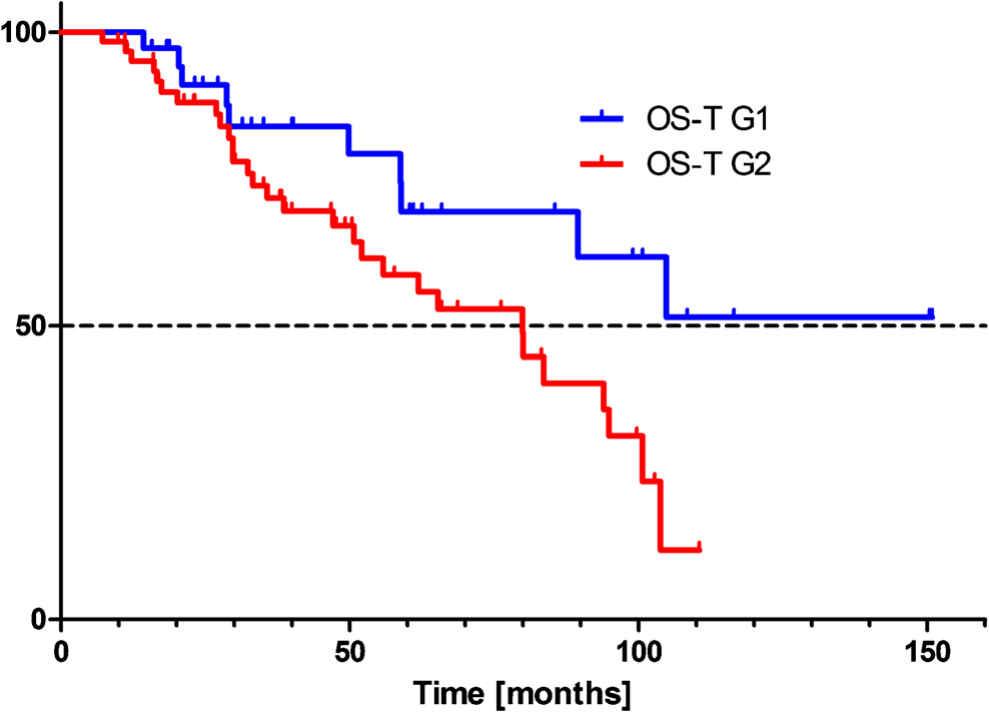
Tandem ^90^Y/^177^Lu-DOTATATE therapy: Kaplan-Meier estimators of overall survival from the time of PRRT (OS-T) in relation to the disease grade

Median OS-T for patients with G2 tumors was 79.9 months, while the median OS-T for patients with G1 tumors was not reached (*P* = 0.026) (Fig.3).

The median overall survival time from diagnosis (OS-D) was 102.6 months for G2 tumors, and not defined for G1 tumors. This difference was not statistically significant on the Kaplan–Meier curve.

**Fig. 4:**
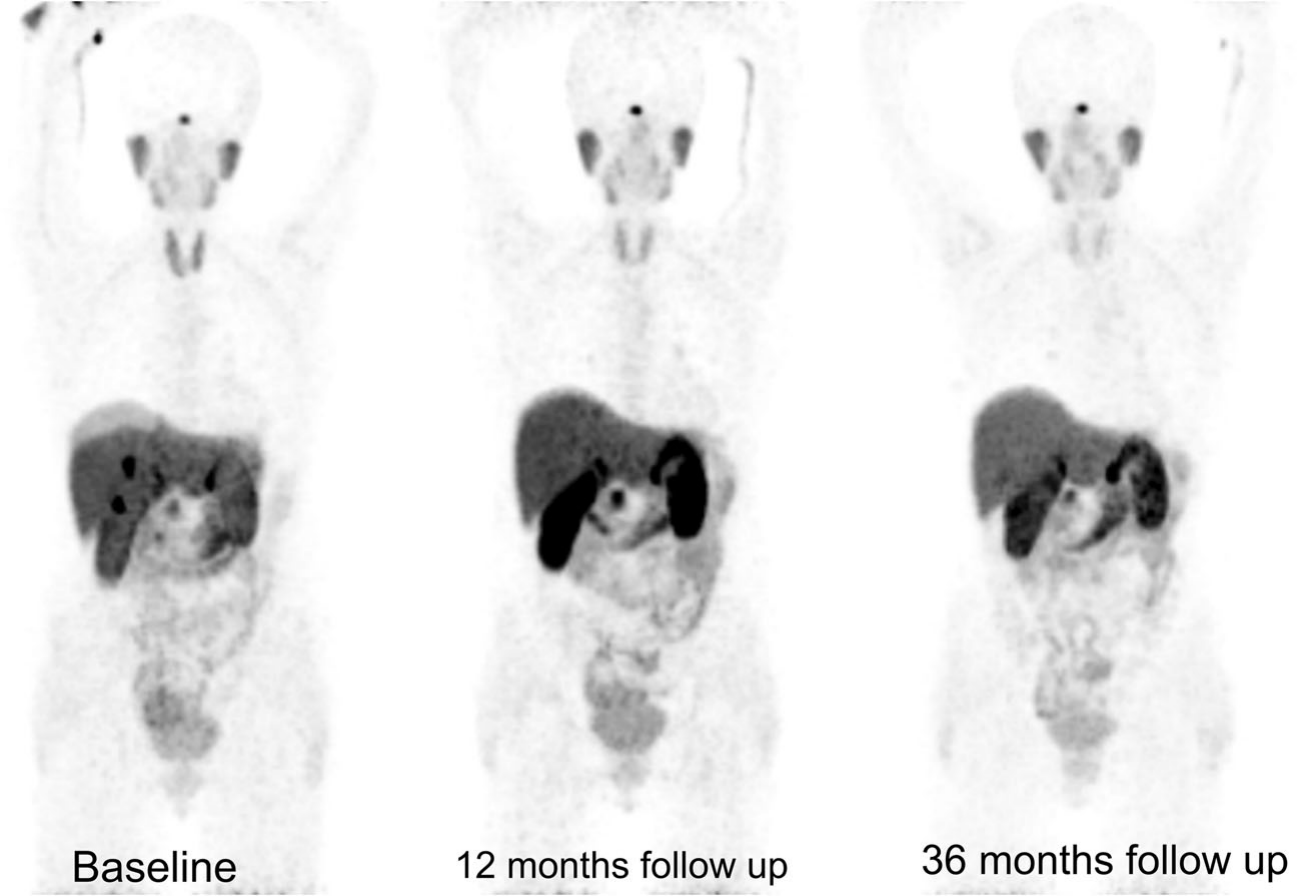
Example of tandem ^90^Y/^177^Lu-DOTATATE therapy effect. A 62-year-old woman with non-functional pancreatic NET G2 and multiple liver metastases, after surgical treatment. ^68^Ga- DOTATATE PET/CT before treatment showing uptake in the metastatic lesions in the liver. At 12 and 36 months of follow up, ^68^Ga- DOTATATE PET/CT showed complete treatment response within liver metastases. Additionally, uptake in the uncinate process of the pancreas is visible, with no abnormality by endoscopic ultrasound (EUS).

**Fig. 5:**
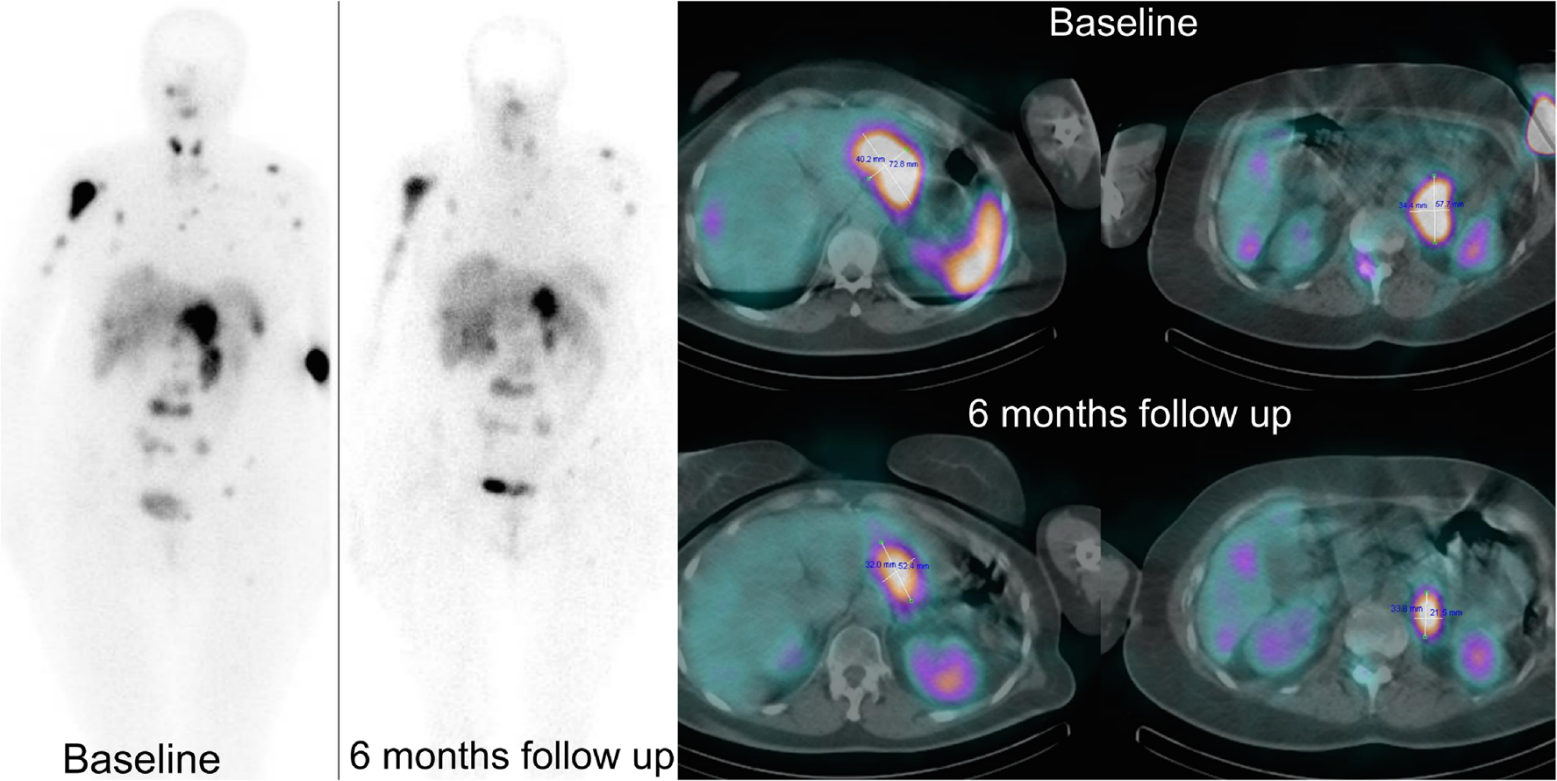
Example of tandem ^90^Y/^177^Lu-DOTATATE therapy effect. A 63y-old woman with non-resectable, non-functional pancreatic NET G1 with metastases to lymph nodes, bones and the liver. ^99m^Tc-HYNIC-TOC before treatment with high uptake in the primary pancreas tumor and in the metastatic lesions as well. Six- month follow up with ^99m^Tc-HYNIC-TOC showed partial remission in the primary tumor and retroperitoneal lymph nodes.

The observed overall 2-year survival from start of PRRT in G1 vs G2 patients was 91% vs 88%, and 5-year survival was 69% vs 56%, respectively. The 2-year risk of progression was 33% in G1 patients and 51% in G2 patients. Examples of therapeutic effect are presented in Figs. 4 and 5.

### Effect of primary tumor site

The effect of the primary disease site was also analyzed. Tumors were -grouped into those arising from the pancreas, small bowel, and large bowel primary sites.

### Pancreatic NET

For pancreatic NET (*n* = 31) PFS was 30.8 OS-T was 94 months and OS-D was 130.4 months.

The 2-year survival from start of PRRT for pancreatic NET was 84% and 5-year overall survival was 43%.

The 2-year risk of progression for pancreatic NET was 45%.

### Small bowel NET

For small bowel NET (*n* = 28) PFS was 30.3 months, and OS-T was 61.9 months. OS-D was 89.2 months.

The 2-year survival from start of PRRT for small bowel NET was 89%, and 5-year overall survival was 29%.

The 2-year risk of progression for small bowel NET 46%.

### Large bowel NET

For large bowel NET (*n* = 20), PFS was 40.6 months, and OS-T was 131.2 months. OS-D was not reached.

The 2-year survival from start of PRRT for large bowel NET was 100%, and 5-year overall survival was 50%.

The 2-year risk of progression for large bowel NET was 45%.

### The survival parameters depend on primary tumor location

A statistically significant difference in OS-T was observed between primary tumors located in pancreas vs large bowel (*p* = 0.033) and small bowel vs large bowel (*p* = 0.009); in OS-D small bowel vs large bowel NET (*p* = 0.047). The difference between other locations and survival parameters was not statistically significant.

The effect of the primary disease site and grading revealed statically significant difference in OS-T for pancreatic NET G1 and G2. Effects of primary tumor site and grading are presented in Tables 2, 3 and 4.

**Table 2:** Survival parameters for pancreatic NET G1 and G2

	Pancreatic NET G1 *n* = 10	Pancreatic NET G2 *n* = 21	p
PFS	nr	29.9	ns
OS-T	nr	55.8	0.04
OS-D	nr	101.3	ns

*nr - not reached

*ns – not statistically significant

**Table 3:** Survival parameters for small bowel NET G1 and G2

	Small bowel NET G1 *n* = 11	Small bowel NET G2 *n* = 17	p
PFS	62.7	27.5	ns
OS-T	89.5	31.0	ns
OS-D	109.4	76.8	ns

*ns – not statistically significant

**Table 4:** Survival parameters for large bowel NET G1 and G2

	Large bowel NET G1 *n* = 10	Large bowel NET G2 n = 10	p
PFS	53.7	38.0	ns
OS-T	nr	83.6	ns
OS-D	nr	134.8	ns

*nr - not reached

*ns – not statistically significant

### Toxicity of ^90^Y/^177^Lu-DOTATATE **tandem therapy**

Treatment was well tolerated by patients and no severe adverse events occurred.

The most common transient acute side effects were nausea (in approximately half of the patients) and asthenia. No patient had a carcinoid crisis following the treatment or any acute reaction during radionuclide therapy infusion.

During the treatment, a transient decrease in platelet and WBC count was observed in three patients (3%).

After completing PRRT, toxicity grade 1 leukocytopenia was found in 17 patients (18%) and grade 2 in two patients (2%); grade 1 thrombocytopenia in 5 patients (5%) and grade 2 in two patients (2%); grade 1 decrease in hemoglobin level was seen in 12 patients (13%) and grade 2 in five patients (5%). One patient (1%) developed myelodysplastic syndrome (MDS). Possible predisposing risk factors for MDS included: previous 2 lines of chemotherapy and high tumor load with hepatic, lymph node and bone metastases. No other grade 3 and 4 hematotoxicity was observed.

Patients who experienced a degree of nephrotoxicity had at least 2 risk factors (diabetes, hypertension, age, previous chemotherapy, chronic NSAID treatment). Only 3 of them had normal kidney function before therapy. Grade 1 nephrotoxicity was seen in 14 patients (15%), and grade 2 nephrotoxicity was seen in 3 patients (3%). No Grade 3 or 4 nephrotoxicity was observed.

To date, no hepatic toxicity of any grade or other clinically significant toxicity has been observed.

## Discussion

PRRT using radiolabeled somatostatin analogs initially with ^111^In, afterwards ^90^Y and now ^177^Lu has been used for more than 20 years. Numerous phase I and phase II studies have shown favorable PFS and OS in NET patients compared to historical controls [9–14].

The NETTER-1, first phase III multicenter, stratified, open, randomized, controlled, parallel-group study demonstrated that [^177^Lu-DOTA,Tyr3]octreotate significantly improved PFS compared to Octreotide LAR (Sandostatin® LAR; Novartis; 60 mg) in patients with advanced midgut NET. The estimated PFS in the ^177^Lu-DOTATATE plus Octreotide LAR group was 40 months vs 8.4 months in the Octreotide LAR arm [16] which is comparable with reported data from non-randomized trials.

These results clearly showed that patients with midgut NET treated by PRRT with ^177^Lu-DOTATATE had a survival advantage compared to other forms of treatment including targeted therapy with mTOR or kinase inhibitor [24, 25].

Research continues into the use of ^90^Y and ^177^Lu, both as single agents and in combination for PRRT. ^90^Y and ^177^Lu have different physical properties, such as the half-life and energy of the β-particles.

Based on Marion de Jong’s study, we used a 50:50 combination of ^90^Y and ^177^Lu activity for tandem PRRT. This protocol was described in 2006, and continued later [17–20].

Recently, the size of individual lesions and optimal radionuclide choice was investigated in dosimetry studies and models. The radius within which 90% of energy is deposited in 5.82 mm for ^90^Y and in 0.62 mm for ^177^Lu, clearly suggest that ^90^Y is not an adequate isotope for eradicating small or micrometastases [26]. ^177^Lu delivers significantly higher normalized absorbed doses to 1-cm, 1-mm and 100-μm lesions than ^90^Y (152 Gy vs 96 Gy, 104 Gy vs 13.3 Gy and 24.5 Gy vs 1.36 Gy, respectively) [26]. However, the dose delivered by ^90^Y increased rapidly when sphere size increased [26].

The optimal composition of isotopes was investigated. The composition for tandem therapy based on 50% ^90^Y and 50% ^177^Lu is probably not the best choice. In one Polish center, proportion 1.85 GBq of ^90^Y and 5.55 GBq of ^177^Lu was examined, but it resulted in significant nephro- and hematotoxicity (not published data).

Based on dosimetry studies, a combination of 2 GBq ^90^Y-DOTATOC and 4 GBq ^177^Lu-DOTATOC seems to be better for patients with larger and moderate size lesions [27].

Consequently, in patients with tumors of various sizes and heterogeneous receptor distribution, the use of radionuclide combinations may lead to higher efficacy compared to the use of a single radioisotope [18, 27, 28].

There are currently no randomized trial data comparing the outcomes of the ^90^Y and ^177^Lu PRRT combination to outcomes after treatment with either of those isotopes alone.

Consequently, though potential efficacy has been reported, the benefit of this combination of radionuclides in humans with NET requires further study [19, 20, 29–32],

Our early results of tandem therapy are encouraging, showing longer overall survival after the combination ^90^Y/^177^Lu -DOTATATE treatment compared to the group treated with ^90^Y-DOTATATE alone. The overall survival for ^90^Y-DOTATATE was 26.3 months and for ^90^Y/^177^Lu -DOTATATE was not reached [19]. Long-term follow-up after tandem ^90^Y/^177^Lu -DOTATATE therapy showed PFS of 32.2 months and OS of 82 months [20]. The simultaneous use of ^90^Y/^177^Lu –DOTATATE in a multicenter study showed PFS 29.9 months and OS 89.5 months.

The use of the combination of both radionuclides has been corroborated by other researchers. Seregni et al. tested a much higher injected activity and different proportion of ^177^Lu DOTATATE (5.55 GBq) and ^90^Y DOTATATE (2.6 GBq) which induced better objective responses in 42.3% of patients, but shorter median PFS 24 months by comparison with our study [33]. Villard et al. and Dumont et al. investigated a different somatostatin analog, DOTATOC. Both groups showed similar and comparable results to our own findings with longer OS after treatment with ^90^Y/^177^Lu-DOTATOC vs ^90^Y-DOTATOC (in the cohort study, OS was 66.1 vs 47.5 months, and in patients with gastrinoma, OS was 60.2 vs 27.0 months, respectively) [29, 30]. However, both authors use combination therapy utilizing alternating cycles of ^90^Y-DOTATOC and ^177^Lu-DOTATOC, not using both isotopes simultaneously.

2 papers have been published in the literature regarding studies with three treatment arms, comparing ^90^Y and ^77^Lu alone to the combination of both radionuclides [31, 32]. Neither of these was a randomized study.

A German multicenter study showed reduced PFS and OS in a group of patients treated with ^90^Y or ^177^Lu only compared to the combination of ^90^Y with ^177^Lu as combined or sequential therapy [31].

Superior response to combination therapy was confirmed in a multivariate survival analysis, in which ^90^Y-DOTATOC plus ^177^Lu-DOTATOC treatment (combined or sequential) was correlated with a longer survival compared to ^90^Y-DOTATOC (66.1 vs 47.5 months; *n* = 1358; *p* < 0.001) or ^177^Lu-DOTATOC alone (66.1 vs 45.5 months; *n* = 390; p < 0.001) [34].

In a recently published paper in 1048 patients treated with ^90^Y and ^177^Lu either as a combination of both radionuclides in one cycle (TANDEM) or sequentially (DUO), or with ^90^Y and ^77^Lu alone, best OS (66 months) was achieved with the combination of ^90^Y and ^177^Lu for PRRT. Treatment with ^177^Lu alone resulted in the next longest survival time (44 months) while the shortest time of survival (24 months) was observed in patients treated only with ^90^Y. The PFS achieved for these 3 patient groups was 24, 17 and 7 months, respectively [32]. Despite smaller group sizes, we obtained similar or even better survival results.

The survival gain we have observed in our study may reflect the relatively high number of NET G2 subjects in our patient population. . In our study,the proportion of G1/G2 was 37%/63% in comparison to the NETTER-1 study where the proportion G1/G2 was reverse 66%/34% [16]. In patients with G2 tumors, ^18^F-FDG PET/CT imaging is a good prognostic indicator, but this is not reimbursed for NET patients in Poland [35, 36].

In our group of patients, we observed better PFS and OS-T for NET G1 patients. This is contrary to observations published by Horsch et al. [31] but consistent with Baum et al. who reported longer overall survival in NET G1 patients and concluded that tumor grade was a strong predictor of overall survival [32].

Published articles had limited data concerning the results of the PRRT in correlation with the origin of primary tumor. Horsch D et al. observed that patients with small bowel NET were significantly less likely to die than patients with other primaries, whereas there was no significant difference in the PFS [31]. The same result was obtained by Baum et al. where the small intestine location had longer OS than NETs arising from other primary sites. [32]. In our previous single center analysis and now from our multicenter study, we observed the longest PFS and OS-T in NET arising from the large bowel [20]. Patients with midgut tumors according to results of PROMID and CLARINET studies, are first treated for a long time with long acting somatostatin analogues. Patients with a other NET localization are more likely to induce to more aggressive treatment including the PRRT. This can have significant effects on survival parameters.

From the clinical point of view, treatment related toxicity is an essential consideration. Published studies have reported several serious side effects of PRRT.

Before the introduction of amino-acid infusion, nephrotoxicity was common and was dose limiting, particularly in association with ^90^Y labeled peptide therapy...

In the NETTER-1 trial with ^177^Lu-DOTATATE, no evidence of renal toxic effects were reported during 14 months follow-up. Nephrotoxicity is a well-known side-effect, requires a minimum follow-up of 6 months to be observed, more likely 1 year [12, 13, 37–39]. In our group treated with tandem ^90^Y/^177^Lu-DOTATATE, grade 3 and 4 toxicity was not observed, even with longer follow-up (median 44.1 months) [16].

Hematological toxicity is usually transient. In the NETTER-1 trial, grade 3 or 4 neutropenia, thrombocytopenia, and lymphopenia were reported in 1%, 2%, and 9% of patients, respectively [16]. In our group, long-term hematotoxicity in the form of MDS was noted in one patient (1%), which is comparable to the results reported in the literature [15, 37]. No other grade 3 and 4 hematotoxicity was observed. No hepatotoxicity or others serious adverse events were noted.

The present study has some strengths and limitations. The main limitation of the present study is its retrospective design, relatively small patient cohort and lack of dosimetry.

Seregni et al. [28] reported the results of a dosimetry phase II study in a group of patients refractory to conventional therapy. By comparison with our own study, higher administered activities of ^177^Lu DOTATATE (5.55 GBq) and ^90^Y DOTATATE (2.6 GBq) did not result in kidney damage and the cumulative BED values were below the toxicity limit in the majority of patients [33]. The dosimetry implications related to the size of the lesion and possible kidney or bone marrow toxicity are certainly important but are not the only variables to consider. Recent published studies have shown that pre existing risk factors for kidney damage increase the probability of PRRT toxicity, so this is a group of patients who might benefit from pre treatment dosimetry studies. On the other hand, dosimetry studies have demonstrated that not only total administered activity per cycle but also the total number of cycles play an important role in PRRT toxicity, lower toxicity being associated with lower cumulative activities [37]. In our treatment protocols, we applied half of the former use injected activity of ^90^Y and much lower activity of ^177^Lu in smaller cohort. Nevertheless, we obtained similar survival rates with a low incidence of side effects.

Limited dosimetry data that we obtained at the beginning of therapy showed absorbed doses for kidney and bone marrow comparable to other publications (for kidney 2.6Gy/GBq for ^90^Y and 0.7 Gy/GBq for ^177^Lu; for bone marrow below 0.2 Gy per cycle) [33, 38]. On the basis of theses reassuring data and busy day clinical practice, this investigation was not continued.

The low incidence of serious adverse events, high probability of disease control and long PFS and OS constitute real benefits for NET patients. However, a limited population size, lack of control group (treated with yttrium or lutetium alone) and lack of randomization will inevitably affect the strength of our conclusions.

The NETTER-1 trial strongly confirmed the effectiveness of PRRT, but the optimal radiolabel and role of combination therapy remains controversial. There is no randomized three-arm trial evidence comparing outcomes for simultaneous ^90^Y/^177^Lu-DOTATATE or sequential ^90^Y- and ^177^Lu-DOTATATE treatment compared to single isotope [31, 32]. This suggests that it need future evaluation in lager group of patients.

## Conclusions

These results of a multicenter trial indicate that tandem radioisotope ^90^Y/^177^Lu-DOTATATE therapy for patients with metastatic neuroendocrine tumors (G1 and G2) is highly effective and safe, with limited side effects. We have also demonstrated statistically significant favorable outcomes of tandem ^90^Y/^177^Lu-DOTATATE therapy in patients with NET G1 compared to NET G2.
